# Effect of education based on the Common-Sense Model of Self-Regulation on blood pressure and self-management of hypertensive patients: A clinical trial study

**DOI:** 10.1016/j.ijnss.2023.06.009

**Published:** 2023-06-20

**Authors:** Zohre Kordvarkane, Khodayar Oshvandi, Younes Mohammadi, Azim Azizi

**Affiliations:** a Student Research Committee, School of Nursing and Midwifery, Hamadan University of Medical Sciences, Hamadan, Iran; b School of Nursing and Midwifery, Hamadan University of Medical Sciences, Hamadan, Iran; c Department of Epidemiology, School of Public Health, Hamadan University of Medical Sciences, Hamadan, Iran; d Member of Chronic Diseases (Home Care) Research Center, School of Nursing and Midwifery, Hamadan University of Medical Sciences, Hamadan, Iran

**Keywords:** Health education, Hypertension, Iran, Nurses, Patients

## Abstract

**Objective:**

This study aimed to determine the impact of training based on the Common-Sense Model of Self-Regulation (CSM) on blood pressure and self-management of patients with hypertension.

**Methods:**

This randomized controlled trial study was conducted. Seventy-two hypertensive patients were referred to the Farshchian Hospital clinic in Hamadan from April 2021 to March 2022. Samples were selected and randomly assigned to the intervention group (*n* = 36) and control group (*n* = 36). The intervention group participated in a training program based on the CSM in five sessions of 30–45 min for one month. Phone follow-up was also done once every three days. The control group only received routine clinic education. The clinic’s nurse measured the patients’ blood pressure, and the participants completed the self-management questionnaire before and three months after the start of the study.

**Results:**

A total of 68 participants completed the study. Results showed that before the intervention, there was no statistically significant difference in the mean scores of self-management and its dimensions, systolic, diastolic, and mean arterial pressure between intervention and control groups (*P* > 0.05). However, after the intervention, the mean of systolic blood pressure (116.21 ± 14.52 vs. 128.62 ± 16.88) mmHg, mean arterial pressure (88.03 ± 8.47 vs. 98.11 ± 11.69) mmHg and the scores of self-management and its dimensions among patients in the intervention group were decreased comparison with control group (*P* < 0.05).

**Conclusions:**

Education based on the CSM improved self-management and blood pressure reduction in hypertensive patients, so nurses should use it as an effective educational model.

## What is known?

• Chronic diseases such as hypertension are complex and require effective models to educate patients on managing them.
• However, only some studies have examined the use of these models in practice.

## What is new?

• A study reported that the training program based on the common-sense model of self-regulation could improve hypertension patients’ blood pressure and self-management.

## 1 Introduction

High blood pressure (HBP) is one of the world’s health problems due to its high prevalence and association with cardiovascular diseases [1]. According to the new definition, hypertension is described as systolic blood pressure (SBP) ≥ 130 mmHg and diastolic blood pressure (DBP) ≥ 80 mmHg (1 mmHg = 0.133 kPa) [2]. Global prevalence of hypertension has increased in the past decade due to changes in lifestyle, behavioral risk factors, aging population, and lack of physical activity [2]. Prevalence of hypertension in adults over 25 in Iran is estimated to be 31% and 27% in men and women, respectively [3].

Self-management is considered one of the most effective strategies for managing hypertension [4]. Self-management refers to the actions taken by the patient to manage their health problem with the support of a healthcare professional [5]. Studies show that many high blood pressure control programs have failed due to the lack of specific goals, inability to control emotions caused by the disease, inappropriate planning, and failure to commit patients to self-management [6]. On the other hand, although antihypertensive drugs are known as the primary treatment for hypertension, these treatments alone will not be effective even if patients fully comply and need the education to change their lifestyle [7].

Awareness, control, and treatment of hypertension should be higher worldwide, especially in middle-income countries [2]. Effective education can help patients understand their condition and change their health behavior [8]. In chronic diseases, education is a specific part of the care program that actively involves the patient in their care [9]. The Common-Sense Model of Self-Regulation (the “Common-Sense Model”, CSM) is an educational model that improves patients’ health behavior [10].

According to this model, each patient would form a system of beliefs about their illness. After perceiving the stimulus, they would interpret the perceived signs and symptoms based on that cognitive pattern. This model states that a patient’s reaction to a threat is determined based on their perceptions and beliefs about six variables: causes, nature, consequences, duration of the illness, manageability, and disease severity. This model also highlights the importance of evaluating the signs and symptoms of the disease and believes that people’s mental representations of the disease are formed based on their perception and awareness, including the perception of a threat, which can affect their behavior [11,12].

Leventhal believes that patients’ decisions while evaluating the signs and symptoms are affected by the perception of the signs and symptoms and their emotional features. Thus, patient’s reactions could differ according to their perception of the character of the threat and cause of the signs and symptoms, their beliefs about the possible consequences, their perception of the severity of the disease, as well as their expectations regarding the treatment and manageability of the signs and symptoms [11,12].

Acceptance of self-management recommendations by patients with chronic diseases is influenced by mental beliefs caused by disease [13]. When facing the disease, the patient draws their understanding of the threatening situation based on personal experiences, previous information, and cultural factors of society, which will determine how to react to the disease [14]. By modulating the effects of external factors, self-regulation systems provide the basis for internal control of behavior [12]. Zakerimoghadam et al. showed that using the model improved heart failure patients’ self-care by enhancing their understanding of the disease [15]. Also, Fall et al. demonstrated that implementing this model of strengthening diabetes perception improved adherence to treatment and quality of life [16]. In Asgari et al.’s study, implementing this model decreased menstrual distress among adolescent participants [17].

Blood pressure management is essential in preventing the disease’s progression and its complications. However, considering the complex nature of chronic diseases and factors influencing health behaviors on the one hand and taking into account that blood pressure control remains a problem in society on the other hand [6,18], taking into account previous studies that have showcased the favorable impact of education based on CSM in enhancing outcomes for various chronic conditions [16,[19], [20], [21], [22]], it can be inferred that such an approach may prove beneficial for the present context. Therefore, it is necessary to use more effective educational models to improve self-management and blood pressure control. This study aimed to determine the impact of education based on the CSM on blood pressure and self-management of patients with hypertension.

## 2 Methods

### 2.1 Study design

This study was a randomized controlled trial that compared two groups through pre- and post-intervention assessments. It was registered and confirmed in the clinical trial registration center with the IRCT code: IRCT20170123032129N8 at the website of https://en.irct.ir/trial/40605. Clinical trials followed the CONSORT criteria for conducting and reporting parallel group randomized trials.

### 2.2 Study setting and participants

This study involved hypertensive patients who visited the heart clinic of Farshc’s study [23], the difference in mean blood pressure between the two groups was 10, with a standard deviation of 10, an error of 1% (the *Z* score is equal to 2.6), a power of 90%, and a lost follow-up rate of 10%, we calculated the sample size to be 36 people in each group.

Inclusion criteria were: being able to read and write, having primary hypertension, being between 40 and 65 years old (since traveling was hard for older patients), and having suitable physical conditions to participate in training sessions. Exclusion criteria were: changing the treatment protocol (such as medication) and having any acute intractable disorders (such as hypertension crisis, stroke, or cardiac attack). Reasons for dropping out were missing more than one session, dying, or failing to complete the questionnaire.

### 2.3 Ethical considerations

Before the intervention, the main researcher explained the study’s purpose, significance, and method to the patients and informed them about voluntary participation and the unaffected withdrawal principle. Then, the participants signed written informed consent. The corresponding researcher obtained permission from the main designer to use the self-management questionnaire. After completing the study, the researcher provided a training booklet to the control group. However, the intervention group still needs to receive a booklet because they had already received the training according to the program. The ethics committee of Hamadan University of Medical Sciences approved this study with the number IR.UMSHA.REC.1397.405.

### 2.4 Randomization

Patients were consecutively selected from eligible patients referred to the clinic above of hospital and randomly allocated to a control and an intervention group using a random permuted block method. Before data collection, the study supervisor prepared a plan for permuted block randomization using an online number generator (i.e., https://www.sealedenvelope.com/simple-randomiser/v1/lists/). The putative participants were randomly allocated into 12 blocks of 6 to be assigned to a control or an intervention group, 36 in each group [24].

### 2.5 Intervention procedure

Only two nurses are working in the hospital clinic. One teaches the patients, and the other monitor their blood pressure, the same for both control and intervention groups. The same cardiologist treats both groups as well.

#### 2.5.1 Intervention group

The intervention group received training based on the CSM [25] by the main researcher (Zohre Kordvarkane) in the clinic’s classroom, in addition to the regular training of the control group in the clinic. The research team prepared the training content based on evidence-based nursing and got approval from the clinic doctor. Patients used private or public transport to come to the clinic from home for the classes. Training sessions were conducted individually and face to face, once every six days, for 30–45 min each for patients. Caregivers of the patients were not present in these meetings. There were five sessions in total. The main researcher also followed up with the patients by phone every three days for 10 min to ensure they continued with the care program. In the follow-up phone calls, the patients were asked about the care emphasized in the class and what they had done to control and manage their blood pressure in the last three days. Some of the questions were: Did you measure your blood pressure? What was your blood pressure? Do you think this blood pressure is normal? What did you do to lower your blood pressure? Did you take your medication? What steps did you take before using them? How has your diet changed after training? Did you inform your family and friends about your illness? Did you do the recommended exercise? What did you do to reduce your daily stress? Did you have a headache caused by high blood pressure? If yes, what did you do? Patients who had managed their condition properly were encouraged and who had not taken appropriate action received the necessary advice and guidance.

This model taught them about disease cognition in five components: description of nature, symptoms, and causes; consequences; duration; and how to control and care for the disease. The training sessions are detailed below.

**First session.** The researcher established rapport with the patients and explained the study’s objectives, and assessed the patient’s perception of the nature, causes, symptoms, and management of hypertension. A researcher asked the patient the following questions: What is high blood pressure? What is its normal range? What are the causes of hypertension? Which causes are controllable, and which are uncontrollable? What do you do to manage and control your hypertension?

**Second session.** In this session, the researcher corrected the patients’ inappropriate perceptions about hypertension that they had expressed in the first session. A researcher based the corrections on the educational goals they had determined beforehand. Researcher also presented and implemented the necessary training according to these goals. The researcher explained to the patients what blood pressure is, its normal range, what causes it, what its signs and symptoms are, and why it is known as a silent disease because it has no symptoms or signs unless it worsens. Then, researcher discussed with the patients the factors that aggravate the symptoms and signs of hypertension, such as extreme activity, mental and physical stress, improper diet, smoking, and problems in taking medicine. The researcher corrected any incorrect perceptions that the patients had about these factors. Researcher taught the patients how to refrain from intense activities, reduce their daily stress by creating healthy entertainment, stay away from high-stress areas, do and accept tasks within their capacity, and avoid arguments with family members or others. Researcher also trained the patients to use low-salt and low-fat foods and add Mediterranean foods to their diet to control their hypertension.

**Third session.** After reviewing previous session topics, the patient’s perception of consequences, duration, and how to control and treat the disease were discussed, and incorrect items were revised. In this session, they learned about blood pressure complications, such as heart attack, stroke, and kidney dysfunction if the disease is uncontrolled. They also learned about the chronicity of the disease, the need to follow up and continue the treatment, self-care, and lifestyle changes to deal with the disease.

**Fourth session** Information from the previous sessions was reviewed to reinforce it. Patients’ perception of drug therapy was evaluated, and oral and written information was provided to correct misconceptions. Finally, a general care plan was presented to each patient. Patients were taught to recognize the symptoms of high blood pressure and its complications and to anticipate their occurrence. Emotional issues were also addressed, and the patients were asked to share their feelings during a normal day and when blood pressure increased and caused complications. Patients were assisted to become aware of their emotional responses to the disease and to report any symptoms and complications caused by it. Patients were also informed about the importance of treatment adherence and the risks associated with inappropriate psychological reactions, such as denial, ignoring symptoms, lack of follow-up, and non-compliance with treatment and care regimens, leading to delays in controlling the disease and worsening symptoms and signs.

**Fifth session.** Previous content was reviewed, and the patient was asked to explain what they understood about their illness. Inappropriate social and cultural factors affecting the patients’ beliefs were identified and corrected if necessary. In this context, some patients may stop their medication after some time under the pressure of family members or friends or resort to unproven treatments such as using traditional drugs. They may also avoid doing the recommended exercises due to the fear of worsening disease or not following their diet because they are taking blood pressure medications. They were also educated in this regard. Patients were encouraged to inform their family members, friends, and colleagues about their illness, treatment, medications, diet, and care principles so that they could provide necessary support in care, disease control, or in cases of sudden problems.

#### 2.5.2 Control group

The control group received a 10-min training session from the clinic nurse on how to take medications, follow the medication regimen, follow the food regimen, exercise, and avoid extreme stress during each visit. They also received a pamphlet that contained the same information. On average, each doctor’s visit lasted 10 min, mostly devoted to taking a medical history and prescribing medicine, and less was devoted to patient education. Therefore, doctors gave brief 1-min training to each patient. These patients visited the hospital clinic every two months to receive medicine, check, and control blood pressure.

### 2.6 Measures

#### 2.6.1 Patient’s demographic characteristics questionnaire

The patient’s demographic characteristics questionnaire consisted of the following variables: age, body mass index (BMI), gender, marital status, occupation, education, income of per month, family history of hypertension, belief in some cure, medication use, and sport before intervention.

#### 2.6.2 Self-management questionnaire

A questionnaire of self-management behaviors of hypertensive patients was designed by Omid et al. in Iran. This questionnaire has 42 questions in 5 dimensions: self-regulation behaviors, self-monitoring behaviors, response to disease, adherence to medication regimen, and self-care behaviors, with 8, 5, 8, 9, and 12 items, respectively. Based on a 5-point Likert scale, it ranges from never with a score of 1 to always with a score of 5. Questions were scored inversely in the area of adherence to the medication regimen. A higher score indicates high self-management. Its validity has been reported favorably in Omidi et al.’s study, and the reliability of the entire questionnaire has been confirmed with Cronbach’s α coefficient of 0.78 [26]. In the current study, Cronbach’s α coefficient of the questionnaire dimensions was estimated to be from 0.81 to 0.93, and the total score was 0.87.

#### 2.6.3 Blood pressure measurement

We used an Omron-HBP-1120 digital sphygmomanometer made in Malaysia to measure the blood pressure of all patients. A clinic nurse who did not know about the groups measured blood pressure between 8:00–10:00 a.m. in a quiet, comfortable environment, with the patient lying down and relaxed. The patient’s arm was at heart level, with the palm up and the elbow slightly bent. The nurse wrapped the cuff snugly around the upper arm, about 2.5 cm (1 inch) above the elbow. The cuff was not too tight or loose to ensure accuracy [27]. A medical engineer calibrated the device to increase the accuracy of the measurement.

### 2.7 Data collection

After obtaining written consent from eligible participants, data collection was done by the main researcher before the study and after the intervention. Patients’ demographic variables questionnaire and the self-management questionnaire of patients with hypertension were given to the patients, and they completed them as self-reports before the intervention in a class in the clinic. Also, the patient’s blood pressure was measured and recorded by one of the clinic nurses who did not know about the study groups. The intervention was carried out for one month, and after three months from the start of the study, at the next visit of the patients to the clinic, the self-management questionnaire was completed by the patients again, and the same clinic nurse measured their blood pressure.

### 2.8 Data analysis

SPSS 16 software was used for the following statistical analyses: Kolmogorov-Smirnov test to assess the normality of the data, paired *t*-test and independent *t*-test to compare the mean values of two groups, and chi-square test to evaluate the relationship between categorical variables. A *P*-value of less than 0.05 was considered statistically significant.

## 3 Results

**Fig. 1:**
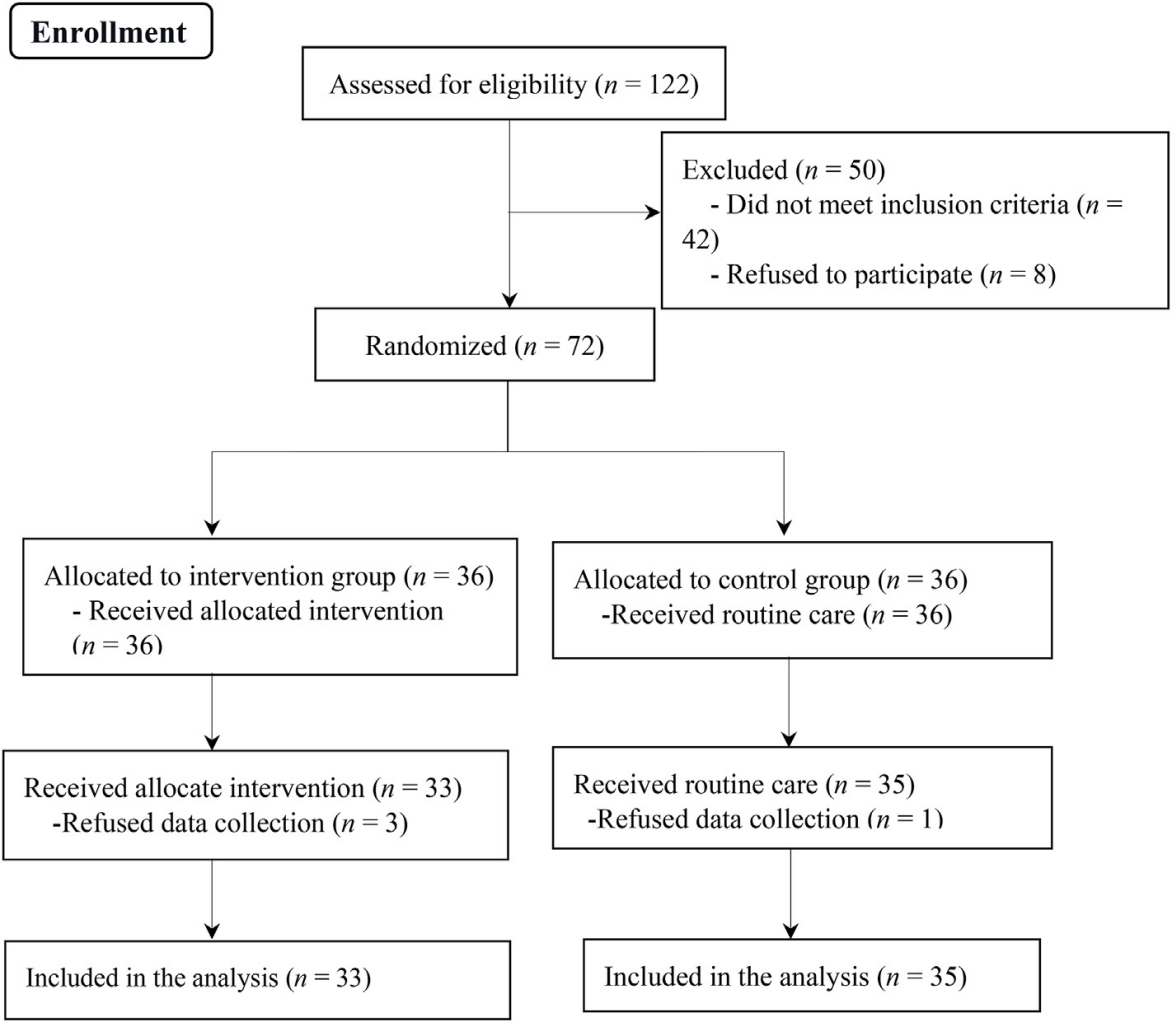
Flowchart of the study.

This study analyzed the data of 68 patients with hypertension. Three people in the intervention group and one people in the control group were refused to participate in data collection (Fig. 1). Overall, the two groups had no difference in baseline characteristics, so the study groups were similar concerning demographic information (*P* > 0.05) (Table 1). Before the intervention, the independent *t*-test result showed no statistically significant difference between the two groups in mean SBP, DBP, mean arterial pressure (MAP), self-care, medication adherence, reaction to the disease, self-monitoring, self-regulation, and total self-management (*P* > 0.05). However, after the intervention, the independent *t*-test revealed a statistically difference between the two groups in mean SBP, MAP, total self-management score, and dimensions (*P* < 0.05). So, SBP and MAP were lower in the intervention group, but self-management and its dimensions were higher than in the control group. Paired *t*-test results demonstrated that there was no statistically change in the control group in the DBP, SBP, MAP, all the dimensions, and total self-management score three months after the study compared to before (*P* > 0.05). However, in the intervention group, DBP, SBP, and MAP scores were decreased, and the total self-management score and its dimensions increased after the intervention compared to before (*P* < 0.05) (Table 2).

**Table 1:** Baseline characteristics of the study group.

Characteristics	Intervention group (*n* = 33)	Control group (*n* = 35)	*t/χ*^2^	*P*
Age, years	55.91 ± 7.44	53.14 ± 8.94	1.382	0.172
BMI, kg/㎡	27.27 ± 4.02	27.51 ± 4.57	0.229	0.819
Gender				
Female	21 (63.6)	20 (57.1)	0.299	0.584
Male	12 (36.4)	15 (42.9)		
Marital status				
Single	1(3.0)	3 (8.6)	1.606	0.527
Married	28 (84.8)	30 (85.7)		
Other	4 (12.1)	2 (5.7)		
Occupation				
Household	11 (33.4)	14 (40.0)	0.71	0.978
Retired	13 (39.4)	12 (34.3)		
Self-employed job	6 (18.2)	7 (20.0)		
Employee	3 (9.0)	2 (5.7)		
Level of education				
Elementary	16 (48.5)	18 (51.4)	0.626	0.731
Diploma	13 (39.4)	11 (31.4)		
College or above	4 (12.1)	6 (17.2)		
Income per month (Dollar)				
≤ 200	3 (9.1)	6 (17.1)	1.212	0.823
201– 400	14 (42.4)	13 (37.1)		
401–600	12 (36.4)	13 (37.1)		
＞600	4 (12.1)	3 (8.6)		
History of hypertension in the family				
Yes	19 (57.6)	21 (60.0)	4.120	0.767
No	14 (42.4)	12 (34.3)		
Belief in some kind of cure				
Taking oral medications	7 (21.2)	8 (22.9)	0.877	0.887
Compliance with the diet	3 (9.1)	2 (5.7)		
Sport	7 (21.2)	10 (28.6)		
All three	16 (48.5)	15 (42.9)		
Taking medication				
Only captopril	7 (21.2)	6 (17.1)	3.120	0.556
Only losartan	8 (24.2)	9 (25.8)		
Losartan and Atenolol	9 (27.3)	6 (17.1)		
Metoral and losartan	5 (15.2)	11 (31.4)		
Another medicine	4 (12.1)	3 (8.6)		
Sport before intervention				
Yes	10 (30.3)	15 (42.9)	0.652	0.419
No	23 (69.7)	20 (57.1)		

*Note*: Data are *n* (%) or *Mean* ± *SD*.

**Table 2:** Comparison of self-management and its dimensions and blood pressure of patients in two groups.

Variables	Intervention group (*n* = 33)	Control group (*n* = 35)	*t*	*P*
Systolic blood pressure (mmHg)				
Pre-intervention	122.48 ± 15.34	127.22 ± 15.93	−1.249	0.216
Post-intervention	116.21 ± 14.52	128.62 ± 16.88	−3.242	0.002
*t*	3.866	−1.284		
*P*	< 0.001	0.208		
Diastolic blood pressure (mmHg)				
Pre-intervention	79.24 ± 9.52	82.28 ± 8.60	−1.384	0.171
Post-intervention	73.93 ± 7.15	82.25 ± 9.72	−4.325	0.163
*t*	3.635	0.702		
*P*	<0.001	0.487		
Mean arterial pressure (mmHg)				
Pre-intervention	93.65 ± 10.50	97.26 ± 10.58	−1.411	0.163
Post-intervention	88.03 ± 8.47	98.11 ± 11.69	−4.084	< 0.001
*t*	4.659	–1.024		
*P*	< 0.001	0.313		
Self-care (12–60)				
Pre-intervention	34.64 ± 5.32	36.28 ± 4.98	−1.319	0.192
Post-intervention	44.21 ± 5.97	36.82 ± 4.58	5.738	< 0.001
*t*	−8.018	−1.866		
*P*	<0.001	0.071		
Adherence to the drug regimen (9–45)				
Pre-intervention	27.54 ± 3.96	26.57 ± 4.53	0.941	0.350
Post-intervention	34.78 ± 3.82	27.17 ± 4.84	7.221	<0.001
*t*	−11.094	−1.558		
*P*	< 0.001	0.128		
Reaction to disease (8–40)				
Pre-intervention	24.06 ± 4.49	23.48 ± 5.34	0.478	0.634
Post-intervention	33.75 ± 3.65	24.94 ± 5.80	7.536	0.001
*t*	−9.011	−1.383		
P	<0.001	0.176		
Self-monitoring (5–25)				
Pre-intervention	23.09 ± 3.64	24.05 ± 5.26	−0.875	0.385
Post-intervention	33.15 ± 3.58	25.08 ± 5.47	7.235	<0.001
*t*	−10.308	−1.024		
*P*	<0.001	0.313		
Self-regulatory (8–40)				
Pre-intervention	12.72 ± 2.84	13.97 ± 4.19	−1.423	0.160
Post-intervention	19.15 ± 5.14	14.22 ± 3.87	3.152	0.003
*t*	−4.656	−1.139		
*P*	<0.001	0.263		
Total self-management (42–210)				
Pre-intervention	122.06 ± 11.45	124.37 ± 14.03	−0.741	0.461
Post-intervention	165.06 ± 12.57	128.25 ± 14.93	10.960	< 0.001
*t*	−14.830	−1.677		
*P*	< 0.001	0.103		

*Note*: Data are *Mean* ± *SD*. 1 mmHg = 0.133 kPa.

## 4 Discussion

Results of the study showed that training based on the CSM reduced blood pressure and improved self-management and all its dimensions, including self-care, medication adherence, response to disease, self-monitoring, and self-regulation of patients with hypertension. In line with the present study, Beaune et al. showed that developing patients’ understanding of the disease, including highlighting the duration of the disease, symptoms, complications, how to control the disease, and social support, could lead to self-control [28]. Zakerimoghadam et al. also concluded that using the CSM by improving the understanding of the disease improved heart failure patients’ self-care [15]. Previous research has found that education based on CSM can increase self-care for patients with tuberculosis [22], improve diabetes perception [19], treatment adherence [20], quality of life [16], and reduce death anxiety in heart failure patients [29], which confirms the findings of the present study.

Using this model to teach hypertensive patients reduced the intervention group’s SBP and MAP compared to the control group. Study results showed that the SBP of the patients in the intervention group decreased from the prehypertensive stage (120–139 mmHg) before the intervention to the normal blood pressure stage (below 120 mmHg). This reduction is clinically significant and indicates that using the CSM has improved the patients’ self-management and perception of the disease, thus positively affecting their blood pressure. Therefore, it is recommended that nurses should include education based on this model along with medication. Leventhal et al. demonstrated that the more patients comprehend the severity of the disease, the more they employ self-regulation strategies to control their condition [25]. In accordance with this research, Taheri-Kharameh et al. showed in a descriptive correlational study that there is a significant relationship between adherence to treatment and perception of the disease in patients with hypertension [30].

Considering the lack of change in blood pressure and self-management in the control group, the clinic’s training could not affect these variables, doubling the need for more effective training. A study showed that blood pressure is diagnosed at any stage, treated in a staged manner, and attention to the disease, self-care, and control decreases over time, consistent with the control group’s results [31]. But in the intervention group, the blood pressure of the patients decreased compared to before the intervention, and the results of the present study are in line with the research results of Najafi Ghezeljeh et al. [32] and Farahmand et al. [33], which are effective in controlling the blood pressure of hypertensive patients.

The study’s results showed that using CSM improved patients’ health behavior with hypertension. This model provided a structure in which the patient understands the risk of the existing disease, the relationship between these conclusions, and how to report the symptoms of the disease so that he can make decisions by improving personal beliefs and self-regulation behaviors. In this study, both the threat recognition and the emotional involvement of the threat were activated to increase the understanding of the disease and self-management. In this study, the patient tried to understand the health threat, get familiar with the symptoms of the disease and his emotional reactions in dealing with the issues related to his disease, understand his vulnerability regarding this disease, and understand the problems related to the disease, try to search for a suitable solution to control and make decisions about their illness, and finally achieve the ability to understand the results of their health-related efforts [34].

Although the prevalence of high blood pressure in Iranian society is higher in men than in women, women go to the clinic more than men. For this reason, the percentage of female participants was higher in selecting research samples, which shows that women are sensitive to their health and attach more importance to it, confirmed by other studies in this field [35].

Results obtained from the study suggest that education based on CSM has the potential to enhance self-management among patients with hypertension. Thus, such teaching programs can affect the perception that directs the patients’ behavior regarding hypertension. Stipulating these convictions can engender superior interventions and foster a healthier lifestyle for the target population. By assessing their self-management perceptions, they can offer subsequent interventions and additional support measures.

## 5 Limitations

The present study employed a sample of literate individuals aged between 40 and 65 years old, so the results of this study cannot be generalized to other people. Implementation of this method requires nurses who have enough motivation and time. Also, because this approach aims to change the patient’s understanding and, ultimately, their behavior, the level of acceptance of the training and their ability to implement the steps are influential factors, and different people need different times for productivity. Furthermore, this study was also constrained by the potential for bias in collecting self-reported data concerning self-management. Patients received personalized training in light of the prevailing circumstances surrounding the COVID-19 pandemic. However, it is advisable to consider group-based training as a viable alternative for optimal utilization of time.

## 6 Conclusions

Study results indicate that training based on CSM with an effect on cognitive and emotional dimensions can improve self-management behaviors and blood pressure control in hypertensive patients. Since this model is an easy, valuable, and practical approach to teaching patients, it is suggested that nurses use this educational method to improve their lifestyle, understand patients, and control and self-manage blood pressure.

## Funding

Acknowledgement financial support for this work was provided by vice-chancellor for research and technology, 10.13039/501100004697Hamadan University of Medical Sciences, Hamadan, Iran. (Grant No. 9806124555).

## Data availability statement

The datasets generated during and/or analyzed during the current study are available from the corresponding author upon reasonable request.

## Declaration of competing interest

The authors declare that they have no competing interests.

## CRediT authorship contribution statement

**Zohre Kordvarkane**: Conceptualization, Methodology, Data curation. **Khodayar Oshvandi**: Data curation, Writing- original draft. **Younes Mohammadi**: Software, Data curation. **Azim Azizi**: Supervision, Conceptualization, Methodology, Writing-reviewing and editing, Visualization.
