# Supplementary material for: Effect of education based on the Common-Sense Model of Self-Regulation on blood pressure and self-management of hypertensive patients: A clinical trial study
Source: Int J Nurs Sci. 2023 Jun 20;10(3):294–301. doi: 10.1016/j.ijnss.2023.06.009 (PMC10401356; doi:10.1016/j.ijnss.2023.06.009)
Supplement: Multimedia component 1 [file mmc1.docx]

基于自我调节常识模型的健康教育对高血压患者血压和自我管理的影响

Zohre Kordvarkane, Khodayar Oshvandi, Younes Mohammadi, Azim Azizi

【**摘要**】

**目的** 本研究旨在确定基于自我调节常识模型（Common-Sense Model of Self-regulation）的健康教育对高血压患者的血压和自我管理的影响。

**方法** 本研究为随机对照临床试验，于2021年4月至2022年3月选取72例转诊到哈马丹Farshchian医院诊所的高血压患者为研究对象，随机分为干预组（*n*=36）和对照组（*n*=36）。干预组患者接受基于自我调节常识模型的培训项目，为期1个月，共5次，每次30～45 min，每3天进行1次电话随访。对照组接受常规健康教育。在干预前、干预后3个月，护士测量患者血压，采用自我管理问卷对患者进行调查，比较两组的干预效果。

**结果** 共有68例患者完成研究。在干预前，两组患者的自我管理得分及其维度得分、收缩压、舒张压和平均动脉压差异无统计学意义（*P*＞0.05）。干预后3个月，干预组患者的收缩压(116.21 ± 14.52 比128.62 ± 16.88) mmHg（1 mmHg=0.133kPa）和平均动脉压(88.03 ± 8.47 比 98.11 ± 11.69)与对照组相比，水平下降，自我管理问卷总得分及其各维度得分均提高，且高于对照组（*P*＜0.05）。

**结论** 基于自我调节常识模型的健康教育改善了高血压患者的自我管理水平，患者血压得到控制，护士可将其作为一种有效的健康教育模式。

【**关键词**】健康教育；高血压；伊朗；护士；病人

通信作者：Azim Azizi, E-mail: Azimazizi1360@gmail.com
